# Optimizing HLA desensitization: serum dilution strategies and platform-specific MFI thresholds for antibody-mediated rejection risk in kidney transplantation

**DOI:** 10.3389/fimmu.2025.1661977

**Published:** 2025-10-02

**Authors:** Dominique Bertrand, Céline Dard, Rangolie Kaveri, Thomas Jouve, Paolo Malvezzi, Lionel Rostaing, Dominique Guerrot, Mathilde Lemoine, Charlotte Laurent, Johan Noble, Fabienne Farce

**Affiliations:** ^1^ Department of Nephrology, Kidney Transplantation and Hemodialysis, Rouen University Hospital, Rouen, France; ^2^ Établissement Français du Sang (EFS), Rhônes Alpes, La Tronche, France; ^3^ Établissement Français du Sang (EFS), Hauts-de-France Normandie, site de Bois Guillaume, Bois guillaume, France; ^4^ Department of Nephrology, Hemodialysis, Apheresis and Kidney Transplantation Department, Grenoble University Hospital, Grenoble, France

**Keywords:** desensitization, HLA, kidney transplantation, luminex, highly sensitized, dilution

## Abstract

**Introduction:**

Highly sensitized kidney transplant (KT) candidates are at increased risk of antibody-mediated rejection (ABMR), particularly in the context of desensitization protocols. Accurate quantification of donor-specific antibodies (DSA) using Luminex single antigen bead (SAB) assays remains essential but is limited by signal saturation and inter-platform variability.

**Methods:**

We conducted a bicentric study involving 20 sensitized KT recipients undergoing HLA desensitization. Anti-HLA antibody profiles were analyzed using Immucor- Werfen (IM) and One Lambda-Thermofisher (OL) SAB platforms. Serum samples were tested both undiluted and at 1:10 dilution before and after desensitization.

**Results:**

Diluted pre-desensitization sera showed stronger correlation with post-desensitization DSA levels than pure sera (R² > 0.90 for IM; > 0.85 for OL). The sum of the DSA (sDSA) outperformed immunodominant DSA (iDSA) in predicting early ABMR. ROC analysis revealed good predictive power (AUC 0.79– 0.88), with IM providing superior iDSA discrimination. For the first time, we propose IM-specific MFI thresholds relevant for ABMR risk stratification.

**Discussion:**

Serum dilution improves the clinical interpretation of DSA intensity and refines risk assessment in desensitized KT candidates. Our findings support the integration of platform-specific MFI cutoffs and dilution-based antibody profiling into organ acceptance strategies for highly sensitized patients.

## Introduction

Kidney transplantation (KT) remains the gold standard treatment for patients with end-stage renal disease, offering significant improvements in survival and quality of life (1, 2). However, sensitizing events such as pregnancies, blood transfusions, or prior transplants can lead to the development of anti-HLA antibodies (3). These antibodies substantially complicate access to transplantation, particularly for highly sensitized patients who remain on waiting lists for extended periods, often without viable donor options (4). Despite the implementation of advanced organ allocation strategies, such as acceptable mismatch programs (5) and priority systems for highly sensitized patients (6, 7), many individuals face persistent barriers due to donor-specific antibodies (DSA) and the difficulty of identifying compatible donors.

To overcome these challenges, HLA desensitization protocols have been developed to reduce antibody levels and improve transplant feasibility. These protocols often include the use of plasmapheresis (PP) (8), intravenous immunoglobulins (IVIg) (9), rituximab (10), and, more recently, innovative therapies such as imlifidase (11, 12). While these strategies have enabled many sensitized patients to undergo transplantation, outcomes remain variable, particularly with regard to long-term graft survival (13–16). Furthermore, desensitization carries an inherent risk of antibody-mediated rejection (ABMR), necessitating improved methods for predicting outcomes and stratifying immunological risk (17).

In this context, advances in anti-HLA antibody detection have been transformative (18). By generating mean fluorescence intensity (MFI) values, Luminex single antigen bead (SAB) assays assays allow semi-quantitative assessment of antibody levels. However, the interpretation of MFI data is complicated by several limitations. These include target saturation, which masks true antibody strength at high titers (19); inhibitory factors, such as complement interference; and shared epitope phenomena (20), which may distribute reactivity across multiple beads, leading to underestimation of immunological risk. These challenges are particularly pronounced in highly sensitized patients, where precise antibody quantification is critical. Serum dilution studies have emerged as a valuable tool to address these limitations. By diluting patient serum, these studies minimize saturation effects and reveal the full spectrum of antibody strength (21). However, the lack of standardized protocols and the variability in assay performance across different commercial platforms, such as Immucor -Werfen (IM) and One Lambda-Thermofisher (OL), complicate the clinical implementation of these findings. A growing body of evidence underscores the importance of platform-specific MFI thresholds to ensure consistent and reproducible interpretations. Studies have shown significant differences in assay design, antigen density, and protocols between vendors, leading to substantial variability in MFI values for the same serum samples (22–24). Consequently, harmonizing MFI thresholds across platforms is essential for clinical decision-making and risk stratification.

This study aims to evaluate the predictive value of serum dilution (1:10) in the context of HLA desensitization. By analyzing diluted serum samples before desensitization process using Luminex SAB technology from the two major platforms (IM and OL), we seek to predict desensitization success and antigen delisting and determine the optimal MFI thresholds to stratify the risk of ABMR. In doing so, this work seeks to provide actionable insights to enhance transplantation success and mitigate immunological risk in this challenging patient population.

## Materials and methods

### Patients

This study included 20 sensitized kidney transplant (KT) recipients from Rouen (n=9) and Grenoble (n=11) University Hospitals. Five received a living donor transplant, and 15 a deceased donor transplant. The desensitization protocol consisted of at least 10 immunoadsorption (IA) sessions over two weeks pre-KT. For living donor transplants, KT was performed after 10 IA sessions, whereas deceased donor recipients continued IA (three sessions per week) until transplantation (median number of IA sessions before transplantation: 32, IQR: 28–44).

All patients received a standardized immunosuppressive regimen: rituximab and antithymoglobulins for induction, followed by maintenance with prednisone, mycophenolate mofetil (1000 mg twice daily), and tacrolimus (initial dose 0.1 mg/kg/day; target trough 8–10 ng/mL). Rituximab was given at a dose of 375 mg/m² (Mabthera^®^, Roche) and rabbit anti-thymocyte globulin at 2.5 mg/kg/day (Thymoglobulin^®^, Sanofi). Patients from two KT centers with available stored sera, who underwent a desensitization strategy prior to KT, were included in this study. Nine patients were from Rouen University Hospital, and eleven patients were from Grenoble University Hospital. In France, highly sensitized candidates (cPRA > 85%) benefit from a national priority system for organ allocation and a structured acceptable mismatch program is implemented. Epitope mismatch data were not routinely available.

Early ABMR after KT was defined as the occurrence of humoral rejection (clinical or subclinical) within the first three months post-transplant. ABMR was diagnosed based on kidney graft biopsy (protocol or for cause) and classified according to the 2019 Banff criteria (25).

In accordance with French law (Loi Jardé: Loi n° 2012–300 du 5 mars 2012), institutional review board approval was not required, as this was an anonymous, retrospective study. The clinical and research activities reported comply with the Principles of the Declaration of Istanbul on Organ Trafficking and Transplant Tourism (26).

### Sera collection and analysis

Serum samples were collected before IA, after 10 IA sessions, on the day of kidney transplantation (KT, Day 0), and at day seven post-transplantation, stored at −80 °C. Retrospective testing was performed using:

- OL Labscreen Luminex Single Antigen (LSA) (Class I/II, depending on antibody presence) at Grenoble University Hospital.
- IM Lifecodes LSA (Class I/II) at Rouen University Hospital.

Mean Fluorescence Intensity (MFI) values were measured for each bead in both pure and 1:10 diluted serum to assess sensitivity and variability between platforms.

To enable platform comparison, the same serum samples from each patient were tested in parallel on both Immucor-Werfen and One Lambda-Thermofisher Luminex SAB platforms, following harmonized protocols and using identical reagent lots. This design ensures within-subject comparison and eliminates potential center-related or inter-patient variability.

### Luminex methodology

Antibody specificity was determined using a Luminex^®^ 200 Instrument (Luminex Corporation, Austin, TX) with LABScreen (OL) and LIFECODES LSA (IM) reagents, following manufacturers’ protocols. To minimize variability, the same lot numbers were used for all sera:

- IM LSA: Class I (3012934), Class II (3012775).
- OL LSA: Class I (13), Class II (15).

### Luminex single-antigen bead assay

#### Immucor-Werfen

Each serum sample (10 μL) was mixed with 40 μL of LSA HLA Class I/II beads and incubated 30 min at room temperature in the dark. After washing, 50 μL of phycoerythrin-conjugated goat anti-human IgG was added, followed by another 30 min incubation. Fluorescence intensity was measured using Luminex 200, and data were analyzed with Match IT! Antibody software. Results were expressed as raw mean fluorescence intensity (MFI). To get rid of any background noise that may falsely positive a bead, a bead‐specific cut‐off based on raw MFI/lowest ranked antigen (LRA) (MFI/LRA) in combination with raw MFI >500 was utilized to assign positive beads. The kit contained 96 Class I beads and 96 Class II beads, with 1 positive and 3 negative control beads.

#### One Lambda-Thermofisher

Test serum (20 μL) was incubated with 5 μL of LABScreen beads. HLA antibodies bound to antigens on the beads were labeled with R-phycoerythrin-conjugated goat anti-human IgG. The Luminex 200 detected fluorescence, and HLA specificity was assigned based on the lot-specific antigen array. Beads with an MFI >2000 were considered positive. The kit contained 97 Class I beads and 95 Class II beads, with 1 positive and 1 negative control bead.

The immunodominant DSA (iDSA) was defined as the DSA with the highest intensity. The sum of the DSA (sDSA) was defined as the sum of the MFI of all beads corresponding to the patient’s potential DSA (A, B, Cw, DR, DQ, DP). For the comparison of the two suppliers, 87 in class I and 67 class II beads, common to both vendors, were analyzed. A total of 1827 class I and 1139 class II identical beads were analyzed, including 120 and 134 DSA beads, respectively.

### Statistical analyses

Quantitative data were reported as mean ± standard deviation (SD) or median with interquartile ranges (IQR) based on distribution. Qualitative variables were expressed as percentages. Given the non-Gaussian distribution of fluorescence intensities, non-parametric tests were used: Wilcoxon signed-rank for paired comparisons (IM vs. OL) and Mann-Whitney U for independent groups. Statistical significance was set at p < 0.05. Pearson and Spearman correlation coefficients assessed linear and rank-based relationships between MFI values, while Bland-Altman plots visualized mean differences (± 1.96 SD). Cohen’s kappa evaluated qualitative concordance between platforms. ROC analysis determined the optimal DSA intensity cut-off for predicting ABMR. Four linear regression models were tested to predict Day 0 KT antibody levels, using different time points (Pure/Diluted Before IA, Combined, and After 10 IA), with performance assessed by R² and mean squared error (MSE). Statistical analyses were performed using GraphPad Prism 8.0 (GraphPad Software, San Diego, CA) and StatView 5.0. (SAS Institute Inc., Brie Comte Robert, France).

## Results

### Correlation matrices of antibody levels across timepoints for HLA class I and class II in IM and OL groups

**Figure 1:**
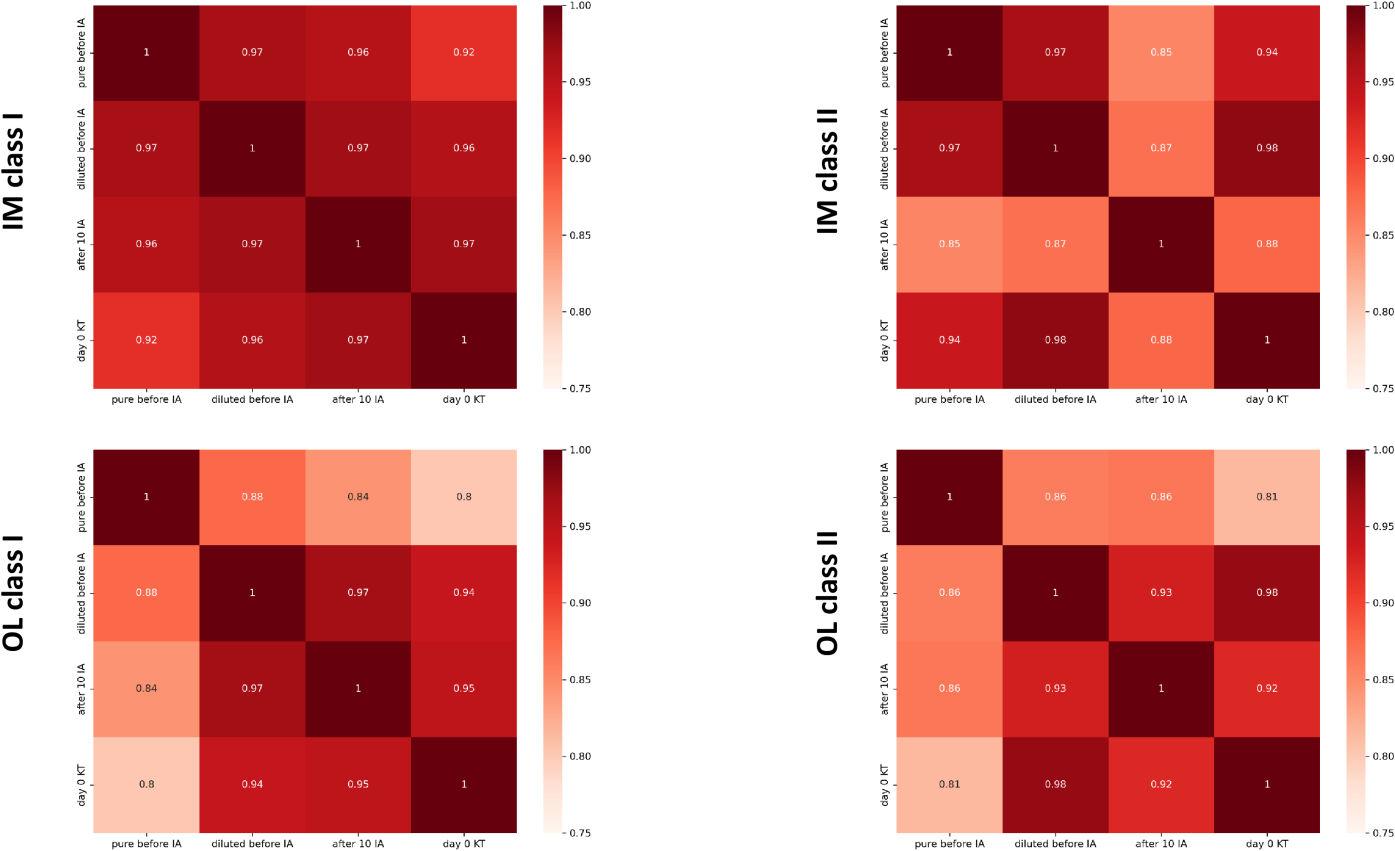
Correlation matrices of antibody levels across timepoints for HLA Class I and Class II in IM and OL groups. The figure presents the correlation matrices showing the Pearson correlation coefficients (RR) between the following antibody level timepoints: pure before IA, diluted before IA, after 10 IA, and day 0 KT. Four separate matrices are displayed: IM Class I: Patients from the IM group, Class I HLA antibodies; IM Class II: Patients from the IM group, Class II HLA antibodies; OL Class I: Patients from the OL group, Class I HLA antibodies; OL Class II: Patients from the OL group, Class II HLA antibodies. The correlation coefficients (RR) are color-coded to highlight positive (red) and negative (blue) correlations. Statistical significance levels are omitted for simplicity (p<0.0001 in all cases). IA, Immunoadsorption.

**Figure 1** illustrates the correlation matrices for HLA antibody levels detected via Luminex Single Antigen assays (IM and OL) across different desensitization and transplantation stages. IM data show highly reproducible measurements, with correlation coefficients exceeding 0.96 between pure before IA, diluted before IA, and Day 0 KT. In contrast, OL data exhibit slightly lower correlations (0.80–0.97), particularly between pure before IA and Day 0 KT, suggesting greater variability in antibody level measurements. Despite both platforms demonstrating statistically significant correlations, IM exhibits stronger consistency across all pairwise comparisons. This highlights potential differences in measurement reproducibility between the two systems.

### Predicting antibody intensity and antigen delisting at day 0 KT using pre- and post-desensitization levels

**Figure 2:**
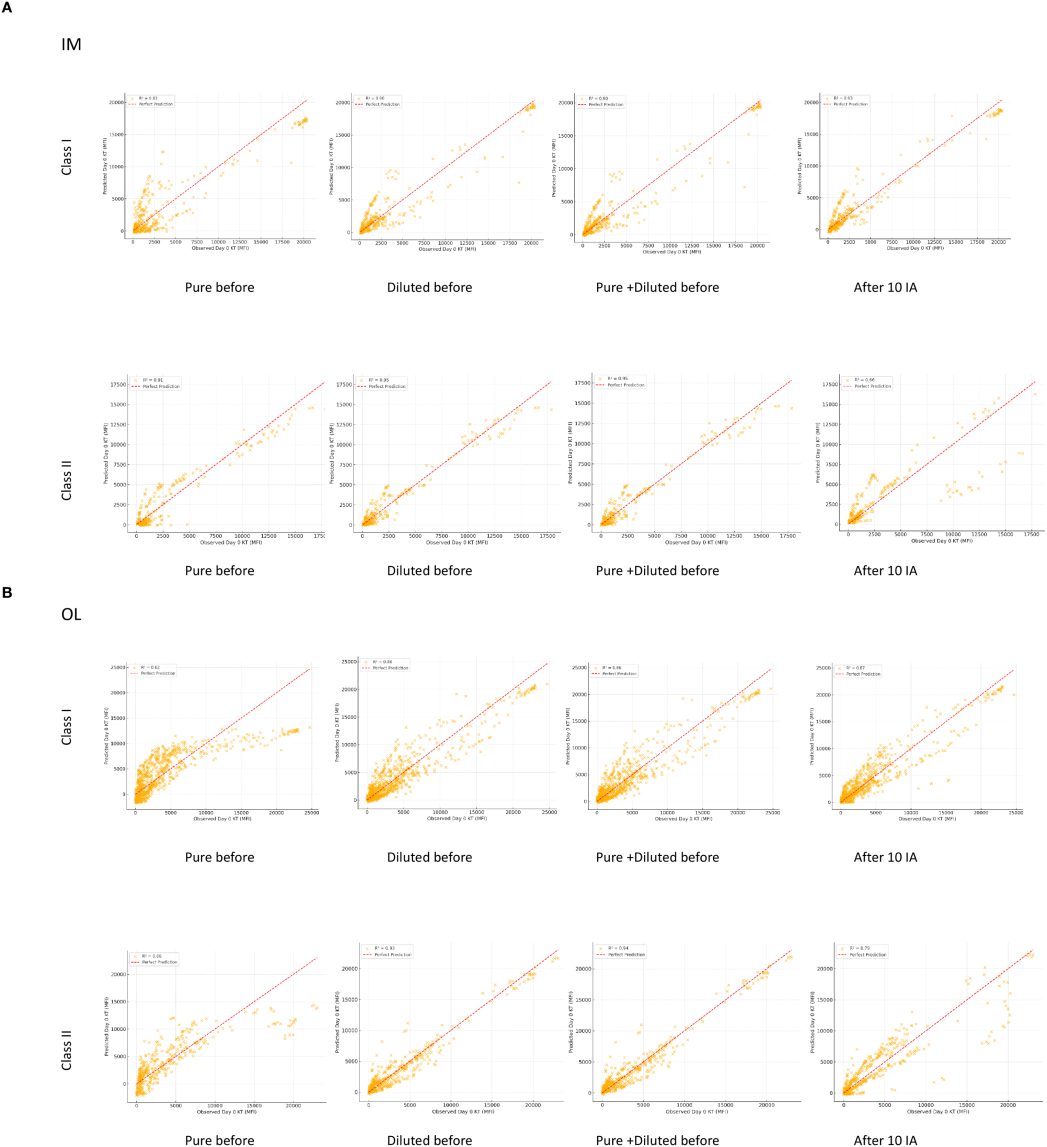
Scatter plots of observed vs. predicted antibody intensity at day 0 KT using different regression models. Each scatter plot illustrates the observed vs. predicted antibody intensity (MFI) at Day 0 KT using one of four linear regression models: Model 1: Pure Before IA; Model 2: Diluted Before IA; Model 3: Combined Pure and Diluted Before IA; Model 4: After 10 IA. The red dashed line represents a perfect prediction (y=x), while the R² value indicates the model’s goodness of fit. Separate plots are provided for each dataset: IM Class I and IM Class II **(A)**, OL Class I and OL Class II **(B)**. The scatter plots are provided for two datasets: **(A)** Immucor (IM)): Results for Class I and Class II and **(B)** One Lambda-Thermofisher (OL): Results for Class I and Class II.

To predict the intensity of antibodies on Day 0 KT and to assist in the possible delisting of antigens before and after the first phase of desensitization, we compared Luminex analysis at different time points using four linear regression models. These results are presented in **Figure 2**. The set of R² and MSE values from the different models are available in **Supplementary Table S1** (**Supplementary Data**).

#### Model performance

Model 4 (After 10 IA) exhibited the highest predictive accuracy across all datasets (**Figures 2A, B**). For instance, in the IM Class I dataset, this model achieved an R² of 0.93 with the lowest MSE, confirming the strong predictive value of antibody levels measured immediately after 10 IA. Model 3 (Pure + Diluted Before IA) outperformed individual pre-desensitization measurements (Models 1 and 2), highlighting the advantage of integrating multiple pre-desensitization data points. Among individual predictors, model 2 (Diluted Before IA) consistently outperformed Model 1 (Pure Before IA), indicating that antibody levels in diluted serum before desensitization provide a better estimation of Day 0 KT intensity.

#### Dataset-specific trends

In the IM datasets, Model 2 (Diluted Before IA) achieved R² values exceeding 0.9, in both class I and II. In the OL datasets, greater variability was observed in the predictive power of pre-desensitization models (Models 1 and 2).

### Comparison of fluorescence intensity and beads positivity between the two luminex suppliers

A total of 87 Class I and 67 Class II beads were common to both Immucor-Werfen (IM) and One Lambda-Thermofisher (OL) platforms.

### Inter-platform correlation

The Inter-platform correlation for class I and class II antibodies results are presented in **Table 1**, which summarizes correlation coefficients, Spearman values, and regression slopes across time points.

**Table 1:** Inter-platform correlation between Immucor-Werfen (IM) and One Lambda-Thermofisher (OL) Luminex SAB assays for Class I and II antibodies across desensitization and transplant stages.

Antibody class	Timepoint	Pearson r	Spearman ρ	Regression slope
Class I	Pure before IA	0.907	0.896	1.290
Class I	Diluted before IA	0.861	0.778	1.302
Class I	After 10 IA	0.910	0.755	1.192
Class I	Day 0 KT	0.871	0.778	1.376
Class I	Day 7 KT	0.896	0.787	1.434
Class II	Pure before IA	0.890	0.780	1.072
Class II	Diluted before IA	0.904	0.705	1.554
Class II	After 10 IA	0.909	0.742	1.539
Class II	Day 0 KT	0.929	0.778	1.170
Class II	Day 7 KT	0.903	0.624	1.252

Correlation analyses between Luminex SAB assays, showing Pearson correlation coefficients (r), Spearman rank correlations (ρ), and regression slopes.

### Inter-platform agreement

**Figure 3:**
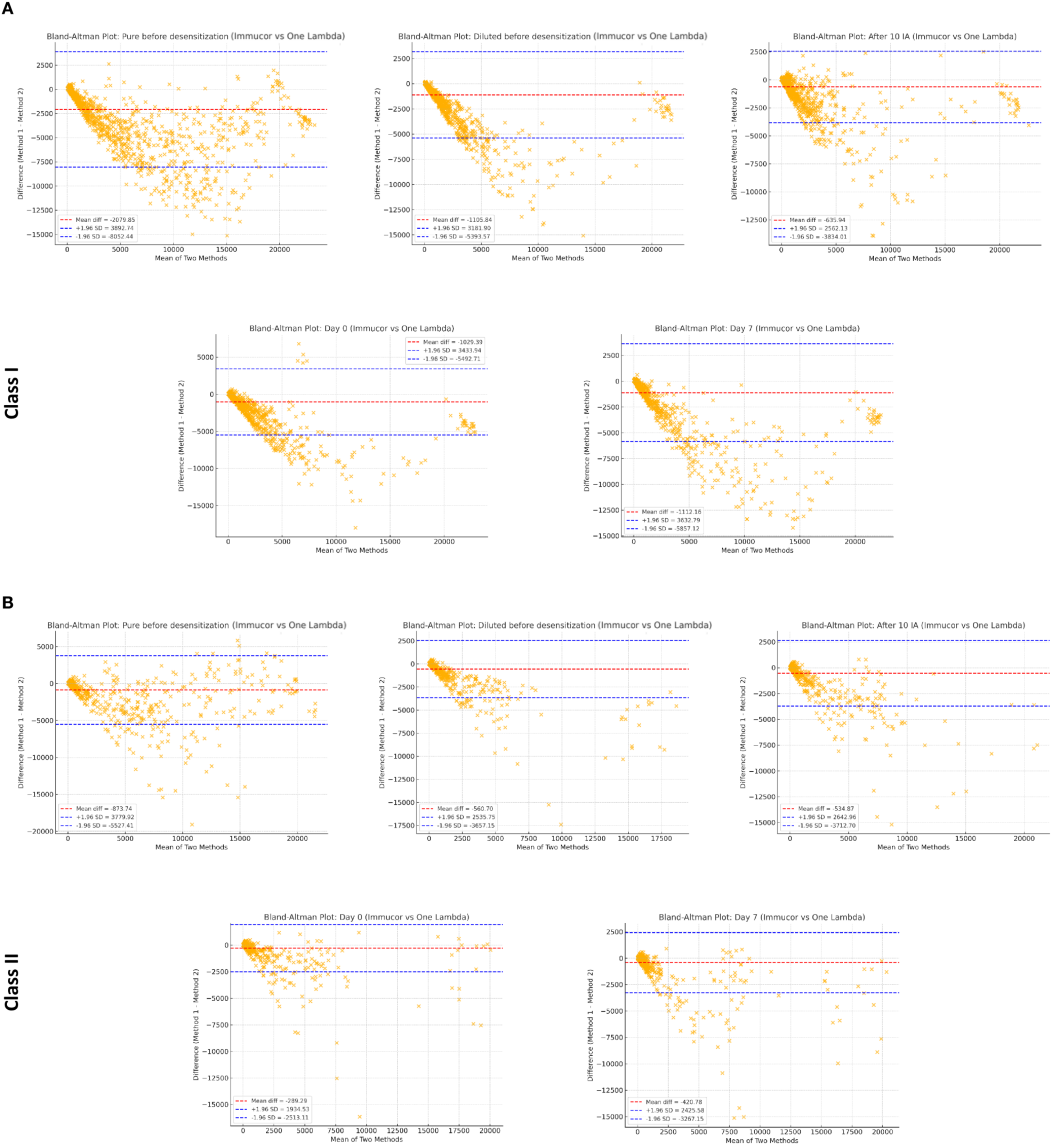
High-resolution Bland-Altman plots comparing Immucor-Werfen and One Lambda-Thermofisher across different sample conditions. Each figure presents high-resolution Bland-Altman plots illustrating the agreement and variability between Immucor-Werfen and One Lambda-Thermofisher measurements under different sample conditions, including pre- and post-transplant phases, as well as before and after desensitization, in class I **(A)** and in class II **(B)**. These plots provide a detailed assessment of method comparability across different experimental settings.

Bland-Altman Analysis (**Figure 3**)

Class I: Systematic biases were observed, but mean differences were close to zero, with acceptable limits of agreement (± 1.96 SD). Greater variability was noted in diluted samples compared to pure serum.

- Class II: Similar systematic biases were present, with higher variability in diluted samples, reflecting differences in calibration and sensitivity between IM and OL.

Cohen’s Kappa Agreement for Positivity Classification

- High agreement was observed at Day 0 KT (κ = 0.802 for Class I, 0.855 for Class II) and Day 7 (κ = 0.853 for Class I, 0.832 for Class II).
- Concordance remained strong before desensitization (κ = 0.895 for Class I, 0.792 for Class II) and after 10 IA (κ = 0.782 for Class I, 0.803 for Class II).
- Lower agreement was observed in diluted samples (κ = 0.604 for Class I, 0.623 for Class II), indicating greater variability between platforms in these conditions.

### Comparison of the evolution of iDSA and sDSA between two luminex suppliers

We compared the evolution of iDSA and sdSA intensity in 20 patients based on the two suppliers, before desensitization (pure serum), on the day of transplantation, and at day 7 post-transplant.

**Figure 4:**
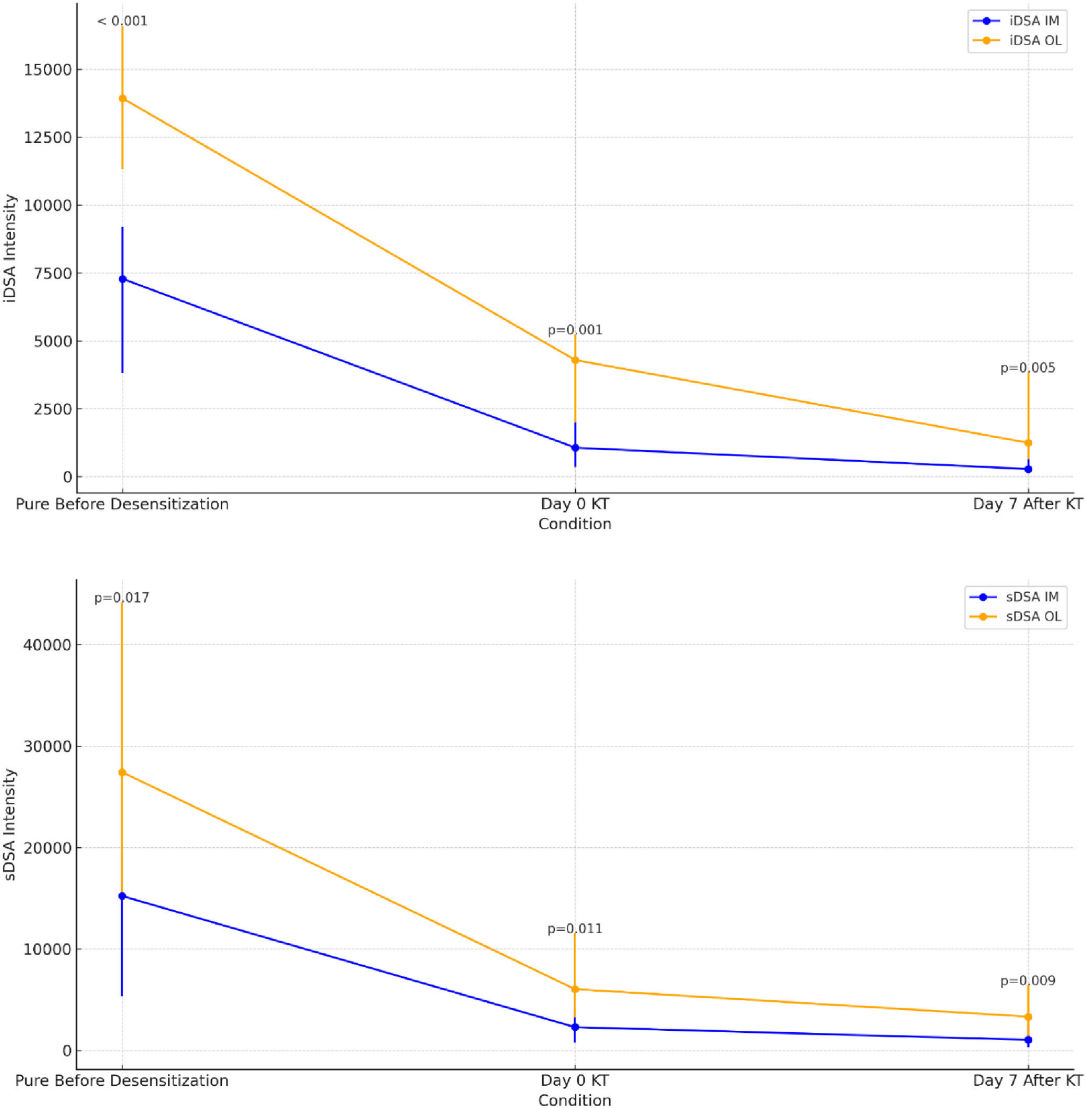
Evolution of iDSA and sDSA intensities measured by Immucor-Werfen and One Lambda-Thermofisher with median, interquartile range, and statistical significance. This figure illustrates the evolution of iDSA (immunodominant donor-specific antibodies) and sDSA (sum donor-specific antibodies) MFI over three conditions: Pure Before Desensitization, Day 0 kidney transplantation (KT) and Day 7 After KT. The intensities are measured using Immucor-Werfen (IM) and One Lambda-Thermofisher (OL). The median values are displayed along with the interquartile range, and statistical significance (p-values) is indicated at each time point to compare differences in intensities.

As shown in **Figure 4**, iDSA intensities varied across conditions, with OL generally exhibiting higher median MFI, particularly at Day 0 and Day 7. The interquartile range was broader for these conditions, indicating variability in antibody detection. Significant differences were observed between the two methods across most conditions. Similarly, sDSA intensities followed a comparable trend, with OL consistently yielding higher median MFI. The statistical analysis confirmed significant differences in antibody intensity between IM and OL, highlighting the impact of assay variability.

**Figure 5:**
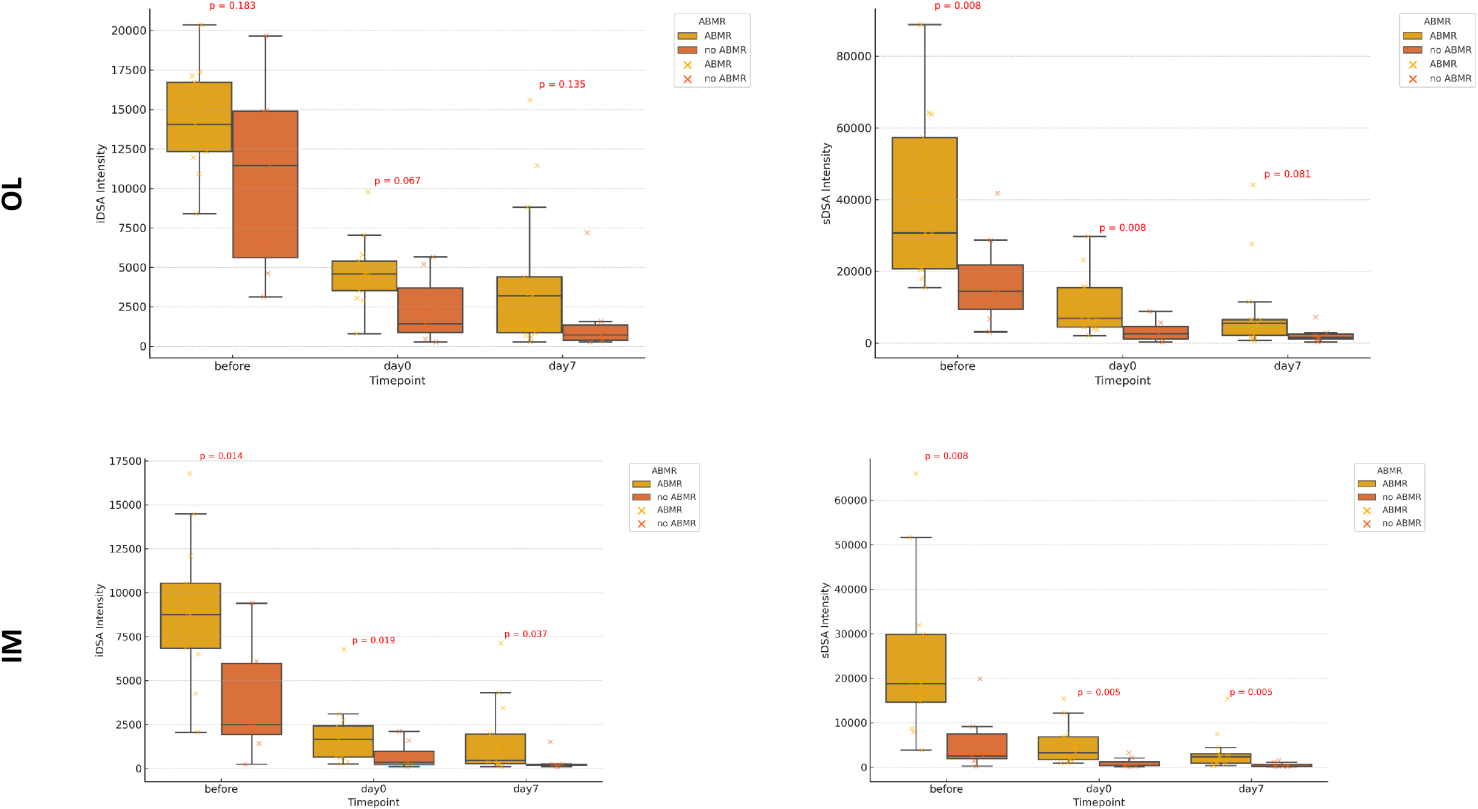
Evolution of iDSA and sDSA intensity over time measured by One Lambda-Thermofisher (OL) and Immucor-Werfen (IM), stratified by the presence or absence of antibody mediated rejection. This figure presents box plots depicting the evolution of immunodominant Donor specific antibody (iDSA) and sum of the DSA (sDSA) intensities at three time points: Before Transplantation, Day 0 kidney transplantation (KT) and Day 7 After KT Stratified by the Presence or Absence of antibody mediated rejection (ABMR). The results are shown separately for: One Lambda-Thermofisher (OL) and Immucor-Werfen (IM). Each box plot illustrates the median, interquartile range, and outliers, with statistical significance (p-values) displayed to indicate differences across time points.

Thirteen patients (13/20: 65%) developed an early ABMR after KT. The incidence of early ABMR was 3/5 (60%) among living donor recipients and 10/15 (67%) among deceased donor recipients. The ABMR group exhibited consistently higher iDSA and sDSA MFI than the no-ABMR group across all time points, regardless of the assay (**Figure 5**). However, statistical significance varied depending on the platform. For IM, iDSA and sDSA values were significantly higher in the ABMR group at all time points. More pronounced differences were observed for sDSA between the ABMR and no ABMR groups at all time points (Before: p = 0.008, Day 0: p = 0.005, Day 7: p = 0.005), suggesting a stronger correlation between sDSA intensity and ABMR risk. For OL, although a trend toward higher iDSA values in the ABMR group was observed, statistical significance was not always reached. While higher median sDSA values were observed in the ABMR group, the difference was not consistently significant across all time points, indicating greater variability in detection between groups.

### Comparison between two luminex suppliers: predictive performance of pre-transplant iDSA and sDSA in assessing ABMR risk in kidney transplantation

Both OL and IM exhibited good predictive performance for ABMR across all time points, including diluted pre-desensitization samples. AUC values ranged from 0.692 to 0.857 for OL and from 0.714 to 0.843 for IM, indicating moderate to strong discrimination (**Table 2**). IM generally achieved higher AUC values for iDSA than OL, suggesting slightly better predictive accuracy. sDSA showed stronger predictive power than iDSA across platforms and time points. AUC values reached 0.857 at multiple time points for both OL and IM, confirming sDSA as a robust ABMR predictor. Diluted pre-desensitization measurements retained good predictive performance (AUC = 0.791 for OL, AUC = 0.857 for IM), with sensitivity and specificity exceeding 0.70 in most cases.

**Table 2:** Predictive thresholds and ROC analysis for ABMR prediction.

Method	Metric	Timepoint	AUC	95% CI for AUC	Optimal threshold	Sensitivity	Specificity	P-value
OL	iDSA	Pure Before Desensitization	0.692	0.393 - 0.920	8389.00	1.00	0.43	0.183
OL	iDSA	Diluted Before Desensitization	0.692	0.381 - 0.920	3039.00	0.92	0.57	0.16
OL	iDSA	Day 0	0.758	0.479 - 1.000	2897.00	0.92	0.71	0.067
OL	sDSA	Pure Before Desensitization	0.857	0.625 - 1.000	15461.00	1.00	0.71	0.008
OL	sDSA	Diluted Before Desensitization	0.791	0.536 - 0.972	5040.00	0.85	0.71	0.04
OL	sDSA	Day 0	0.857	0.641 - 1.000	3726.00	0.92	0.71	0.008
IM	iDSA	Pure Before Desensitization	0.835	0.595 - 1.000	6505.00	0.85	0.86	0.014
IM	iDSA	Diluted Before Desensitization	0.813	0.562 - 0.980	987.00	0.77	0.86	0.02
IM	iDSA	Day 0	0.824	0.573 - 1.000	372.00	0.92	0.71	0.019
IM	sDSA	Pure Before Desensitization	0.857	0.625 - 1.000	7971.00	0.92	0.71	0.008
IM	sDSA	Diluted Before Desensitization	0.857	0.625 - 1.000	1112.00	0.92	0.71	0.01
IM	sDSA	Day 0	0.879	0.667 - 1.000	946.00	1.00	0.71	0.005

This table summarizes the predictive performance of donor-specific antibody (DSA) intensities for ABMR across different metrics (iDSA, sDSA), platforms (One Lambda-Thermofisher; OL, Immucor-Werfen: IM), and timepoints (pre-desensitization pure, pre-desensitization diluted, Day 0). Metrics include area under the curve (AUC), 95% confidence intervals, optimal thresholds, sensitivity, specificity, and p-values for each ROC analysis.

## Discussion

Accurate quantification of anti HLA antibodies is essential for managing highly sensitized kidney transplant candidates (21). Serial serum dilutions improve alloantibody estimation and enhance cPRA assessments in sensitized patients (27). Moreover, pre-transplant serum dilution has been recognized as a useful tool for defining unacceptable antigens and predicting the likelihood of successful desensitization (28). These findings support the use of titration studies to determine whether desensitization strategies, such as PP and IVIg, will be effective in reducing anti HLA antibodies levels below a certain intensity thresholds allowing for the delisting of the corresponding antigens (29): transplant candidates with DSAs of titer ≥1:1,024 at baseline were unlikely to achieve sufficient antibody reduction with PP/IVIg alone. Our findings confirm that 1:10 serum dilution enhances the predictive value of desensitization efficacy compared to pure serum analysis. Bead saturation at high MFI values likely explains this effect, as observed in both OL and IM assays. Dilution provides a more accurate estimation of antibody strength, allowing for improved risk stratification post-desensitization (28). Furthermore, our data suggest that diluted pre-IA samples strongly correlate with Day 0 KT anti HLA levels. An antibody whose intensity in pure serum does not decrease in serum diluted at 1:10 is unlikely to have its corresponding antigen delisted after desensitization. On the day of transplantation, its intensity will be close to that observed in the 1:10 diluted serum before any desensitization procedure has been initiated. This supports the idea that dilution-adjusted MFI values may serve as a biomarker for potential desensitization success. The French Consensus Guidelines on Imlifidase for Kidney Transplantation endorse this approach, recommending 1:10 dilution-based MFI thresholds, where an MFI < 5,000 (OL) is favorable for KT (30).

After desensitization, graft acceptance decisions should be guided by the immunological risk associated with preformed donor-specific antibodies (DSA). In this study, we identified, for the first time, predictive iDSA and sDSA thresholds for antibody-mediated rejection (ABMR). These thresholds enable risk stratification at the time of organ allocation, aiding in transplant acceptance decisions. MFI values from SAB assays are commonly used as surrogate markers for ABMR prediction and graft survival assessment (31). Vo et al. reported the incidence of ABMR in a cohort of 226 highly sensitized patients who underwent transplantation following desensitization. They concluded that the DSA-relative intensity scores at the time of transplant were strong predictors of ABMR (32). Using the OL assay on 432 sera, which were also tested in T-cell crossmatches (XMs), Visentin et al. demonstrated that the SAFB MFI threshold predicting T-cell flow cytometry crossmatch (FCXM) positivity ranged between 4,400 and 6,200 for class I DSA (33). Additionally, the threshold for predicting T-cell complement-dependent cytotoxicity crossmatch (CDCXM) positivity was found to be between 8,900 and 13,600. However, to date, no published data are available for the SAB assay from IM, and the lack of standardized MFI cut-offs across platforms remains a challenge. For the first time, we were able to determine specific MFI thresholds for both OL and IM before desensitization - on both pure serum and 1:10 diluted serum - as well as after desensitization, just prior to transplantation. Both OL and IM exhibited good predictive performance for ABMR across all time points, including diluted pre-desensitization samples. These findings highlight that both suppliers offer strong predictive value for ABMR, with IM showing a slight advantage in iDSA assessment and sDSA emerging as the most reliable predictor of ABMR across all conditions.

This multicenter study is the first to define specific MFI thresholds in the context of desensitization for IM Lifecodes assays. Previous studies have highlighted systematic differences between OL and IM assays in terms of MFI values and sensitivity (22–24), but none have proposed threshold-based risk stratification for desensitization candidates. While both platforms demonstrated strong correlation in bead intensity, our study identified significant variability in absolute MFI values and intergroup discrimination, as already noted in the context of ABMR diagnosis (24). OL consistently reported higher absolute iDSA and sDSA values, whereas IM showed superior ability to differentiate between ABMR and non-ABMR cases. These findings confirm that direct MFI comparisons between platforms are not clinically interchangeable and that assay-specific cut-offs are necessary, not only in routine daily care but especially in the context of clinical trials focusing on this topic. Furthermore, our study is the first to assess desensitization-specific MFI thresholds for IM, a key finding given that most prior research has focused exclusively on OL (17). Recent approaches, such as non-linear modeling, have provided a means to convert MFI values between platforms and establish more reliable thresholds for both class I and class II HLA antibodies (34). Nevertheless, Karahan et al. concluded that there were considerable variations between the two assays and using MFI conversion for individual patient samples was not recommended. The results reinforce the importance of developing vendor-specific risk models to improve clinical decision-making in highly sensitized patients.

This study has several strengths. It is the first to establish MFI cut-offs for IM in the context of desensitization, providing a unique contribution to the field of antibody risk stratification. The large-scale bead analysis, which included 1,827 class I and 1,139 class II beads, provided a robust dataset for cross-platform comparison. Additionally, the bicentric design ensured harmonized testing conditions, with assays performed in the same laboratory, using identical reagent lots, minimizing technical variability. The study also incorporated comprehensive statistical analyses, including ROC curves and predictive modeling, to define platform-specific MFI cut-offs. However, there are some limitations. The study did not assess peak MFI intensity, which has been linked to long-term graft outcomes. Additionally, while the bead dataset was extensive, the main limitation of our study is the small cohort size (n=20), which restricts the generalizability of the proposed thresholds and carries a risk of overfitting. In addition, the lack of external validation precludes any immediate clinical application of the MFI cut-offs we report. These thresholds should be considered as preliminary and exploratory, and require confirmation in larger, prospective multicenter cohorts. Another limitation is the focus on early ABMR as the main endpoint, without medium- or long-term follow-up on graft function or survival. Finally, our assays were performed under controlled laboratory conditions, which may not fully capture the variability encountered in routine multicenter practice.

This study reinforces dilution-based strategies to optimize clinical decision-making in highly sensitized kidney transplant candidates and supports the importance of vendor-specific thresholds. Moreover, it is the first to define specific MFI thresholds in desensitization and establish IM cut-offs for ABMR risk stratification. Future research should focus on harmonizing MFI cut-off values across commercial assays to facilitate standardized risk assessment. Longitudinal studies are needed to evaluate post-transplant DSA kinetics and refine ABMR prediction models. Lastly, evaluating desensitization outcomes with novel therapies such as Imlifidase, particularly with dilution-based risk assessment, could provide valuable insights into optimizing transplant success.

## Ethical compliance

All procedures performed in studies involving human participants were in accordance with the ethical standards of the institutional and/or national research committee and with the 1964 Helsinki Declaration and its later amendments or comparable ethical standards.

## Data Availability

The raw data supporting the conclusions of this article will be made available by the authors, without undue reservation.
