# Supplementary material for: Optimizing HLA desensitization: serum dilution strategies and platform-specific MFI thresholds for antibody-mediated rejection risk in kidney transplantation
Source: Front Immunol. 2025 Oct 2;16:1661977. doi: 10.3389/fimmu.2025.1661977 (PMC12527829; doi:10.3389/fimmu.2025.1661977)
Supplement: Supplementary file 1 [file Presentation1.pdf]

Figure S1. Assessment of the Model performance for the prediction of Antibody Intensity at Day 0 KT Using Pre- and Post-Desensitization Levels using the coefficient of determination (R^2) and mean squared error (MSE).

		R^2	MSE
IM_class_1	Model 1 (Pure Before IA)	0.8204327729319176	4869101.9658649415
IM_class_1	Model 2 (Diluted Before IA)	0.9022101576728114	2651645.9673145483
IM_class_1	Model 3 (Pure + Diluted)	0.9031277760379645	2626764.047274727
IM_class_1	Model 4 (After 10 IA)	0.9340085754326477	1789407.6794386087
IM_class_2	Model 1 (Pure Before IA)	0.9084589723837387	1515963.3003543576
IM_class_2	Model 2 (Diluted Before IA)	0.9510095426414809	811305.4589513605
IM_class_2	Model 3 (Pure + Diluted)	0.951058108919522	810501.1781052483
IM_class_2	Model 4 (After 10 IA)	0.6649220975547336	5549050.694472503
OL_class_1	Model 1 (Pure Before IA)	0.6192415890483475	9992337.978470566
OL_class_1	Model 2 (Diluted Before IA)	0.8608739795246031	3651118.86751283
OL_class_1	Model 3 (Pure + Diluted)	0.8610325211732613	3646958.2194706816
OL_class_1	Model 4 (After 10 IA)	0.8722339120978218	3352997.3226714954
OL_class_2	Model 1 (Pure Before IA)	0.6622808578158268	8122111.943594496
OL_class_2	Model 2 (Diluted Before IA)	0.9346536755507961	1571572.6353155265
OL_class_2	Model 3 (Pure + Diluted)	0.9352954160727924	1556138.8392781313
OL_class_2	Model 4 (After 10 IA)	0.788954887391474	5075614.069930812
